# The evolution of community-based primary health care, Slovenia

**DOI:** 10.2471/BLT.19.239616

**Published:** 2020-03-09

**Authors:** Anne S Johansen, Pia Vracko, Robert West

**Affiliations:** a World Health Organization European Centre for Primary Health Care, Kazakh National Medical University, 88 Tole Bi Street, Almaty, 050012 Kazakhstan.; b National Institute of Public Health of the Republic of Slovenia, Ljubljana, Slovenia.; Correspondence to Anne S Johansen (email: johansena@who.int).

## Abstract

**Problem:**

Slovenia’s model of primary health care relied on reactive, episodic care and was ill-equipped to address the country’s burden of disease dominated by noncommunicable diseases.

**Approach:**

The government has developed a multidisciplinary, community-based, prevention-oriented service delivery model for primary health care. A compulsory family medicine residency programme was introduced in 2000, and from 2004 screening and control of chronic diseases were established in family medicine practices. Health promotion centres were established, providing group interventions to support healthy lifestyles. After 2011, registered nurses were introduced to conduct screening for chronic diseases, provide counselling and manage patients with stable noncommunicable diseases.

**Local setting:**

In 1992, the government transformed Slovenia’s health financing scheme to a social insurance system based on mandatory payroll taxes. The system enabled private provision of health services, although primary care was mostly provided by publicly funded community health centres. A strong gatekeeping role was introduced.

**Relevant changes:**

Despite spending less on health than the European Union (EU) average, by 2013 Slovenia’s life expectancy was higher than the average for EU countries. The increase was due in part to rapidly declining infant and under-five mortality and a faster decline in premature mortality due to chronic diseases.

**Lessons learnt:**

Slovenia’s approach was enabled by strong public health and governance structures, along with accountability mechanisms that monitored outcomes and took corrective action when necessary. New programmes were piloted, creating a strong evidence base that facilitated obtaining sustainable financing, while national roll-out was supported by regional branches of the National Institute of Public Health.

## Résumé

**Problème:**

Le modèle slovène de soins de santé primaires reposait sur une prise en charge épisodique et réactive. En outre, il n'était pas équipé pour faire face au fardeau des maladies du pays, dominé par des affections non transmissibles.

**Approche:**

Le gouvernement a développé un modèle de prestation de services multidisciplinaire, communautaire et axé sur la prévention pour les soins de santé primaires. Un programme obligatoire de résidence en médecine familiale a été instauré en 2000 et, à partir de 2004, un système de dépistage et de suivi des maladies chroniques a été mis en place dans les cabinets de médecine familiale. Des centres de promotion de la santé ont été créés afin d'effectuer des interventions de groupe destinées à encourager l'adoption d'un mode de vie sain. Dès 2011, des infirmières agréées ont été chargées de mener des actions de dépistage des maladies chroniques, de fournir des conseils et de gérer les patients présentant des affections non transmissibles stables.

**Environnement local:**

En 1992, le gouvernement a transformé le régime slovène de financement de la santé en système d'assurance sociale basé sur des taxes obligatoires prélevées sur les salaires. Ce système a entraîné l'apparition de prestations privées de services de santé, bien que les soins primaires soient principalement prodigués par des centres de santé communautaires financés par l'État. Une fonction de contrôle efficace a été établie.

**Changements significatifs:**

Malgré des dépenses en soins de santé inférieures à la moyenne de l'Union européenne (UE), l'espérance de vie slovène en 2013 était plus élevée que celle observée dans les autres pays de l'UE. Cette augmentation s'expliquait en partie par une diminution rapide de la mortalité chez les nourrissons et les enfants de moins de cinq ans, ainsi que par une accélération de la baisse des décès prématurés liés à des maladies chroniques.

**Leçons tirées:**

L'approche de la Slovénie a été rendue possible par de solides structures de gouvernance et de santé publique, mais aussi par des mécanismes de responsabilité qui ont suivi les résultats et pris des mesures correctives le cas échéant. De nouveaux programmes ont été testés et ont engendré une base factuelle stable qui a facilité l'obtention de financements durables, tandis que le déploiement à l'échelle nationale a reçu le soutien des antennes régionales de l'Institut national de santé publique.

## Resumen

**Situación:**

El modelo de atención primaria de la salud de Eslovenia se basaba en el cuidado paliativo y episódico, y no estaba bien equipado para atender a todas las enfermedades del país, que eran en su mayoría enfermedades no transmisibles.

**Enfoque:**

El Gobierno ha elaborado un modelo de prestación de servicios multidisciplinarios, comunitarios y orientados a la prevención para la atención primaria de la salud. En 2000, se adoptó un programa de residencia obligatorio en medicina familiar y, a partir de 2004, se estableció un programa de detección y de control de las enfermedades crónicas en los consultorios de medicina familiar. Se establecieron centros de promoción de la salud, en los que se realizan intervenciones en grupo para apoyar estilos de vida saludables. A partir de 2011, se introdujeron enfermeras tituladas para realizar exámenes de detección de enfermedades crónicas, prestar asesoramiento y atender a pacientes con enfermedades estables no transmisibles.

**Marco regional:**

En 1992, el Gobierno transformó el plan de financiamiento de la salud de Eslovenia en un sistema de seguro social basado en impuestos obligatorios sobre las nóminas. El sistema permitía la prestación privada de servicios sanitarios, aunque la atención primaria se prestaba principalmente en centros de salud comunitarios que se financiaban con fondos públicos. Además, se adoptó una función importante de control de acceso.

**Cambios importantes:**

Aunque el gasto en salud es inferior en comparación con la media de la Unión Europea (UE), en 2013, la esperanza de vida de Eslovenia era superior a la media de los países de la UE. El incremento se debió en parte a la reducción rápida de la mortalidad de infantes y de niños menores de cinco años y a una disminución más rápida de la mortalidad prematura por enfermedades crónicas.

**Lecciones aprendidas:**

El enfoque de Eslovenia fue posible gracias a las estructuras sólidas de salud pública y de gobernanza, junto con los mecanismos de rendición de cuentas que supervisaban los resultados y adoptaban medidas correctivas cuando era necesario. Se probaron nuevos programas, lo que creó una base de evidencia sólida que facilitó la obtención de un financiamiento sostenible, mientras que las filiales regionales del Instituto Nacional de Salud Pública apoyaron la puesta en marcha a nivel nacional.

## ملخص

المشكلة اعتمد نموذج الرعاية الصحية الأولية في سلوفينيا على الرعاية التفاعلية والعرضية، كما كان هذا النموذج غير مجهز لمواجهة عبء الأمراض في الدولة والذي يخضع لسيطرة الأمراض غير المعدية.

الأسلوب قامت الحكومة بتطوير نموذج رعاية صحية أولية لتقديم الخدمات يتميز بتعدد التخصصات، والاعتماد على المجتمع، ويكون موجهًا نحو الوقاية. تم تقديم برنامج إلزامي لطب الأسرة في عام 2000، ومنذ عام 2004 تم إدخال برنامج الفحص والسيطرة على الأمراض المزمنة إلى ممارسات طب الأسرة. كما تم إنشاء مراكز لتعزيز الصحة، والتي توفر التدخلات الجماعية لدعم أنماط الحياة الصحية. بعد عام 2011، تم الاستعانة بالممرضات المسجلات لإجراء فحص للأمراض المزمنة، وتقديم المشورة وإدارة المرضى الذين يعانون من أمراض غير معدية مستقرة.

المواقع المحلية في عام 1992، قامت الحكومة بتحويل مخطط التمويل الصحي في سلوفينيا إلى نظام للتأمين الاجتماعي قائم على الضرائب الإجبارية على الرواتب. أتاح النظام فرصة توفير الخدمات الصحية الخاصة، على الرغم من أن الرعاية الصحية الأولية تم توفيرها في الغالب من جانب مراكز الصحة المجتمعية الممولة من القطاع العام. كذلك ظهر دور قوي للبوابات الإعلامية.

التغيّرات ذات الصلة على الرغم من الإنفاق على الصحة بشكل أقل من متوسط الاتحاد الأوروبي (EU)، إلا أن متوسط العمر المتوقع في سلوفينيا في عام 2013 كان أعلى من المتوسط في دول الاتحاد الأوروبي. وكانت الزيادة ترجع بشكل جزئي إلى الانخفاض السريع في معدل وفيات الرضع والأطفال تحت سن الخامسة، وتراجع بشكل أسرع في معدل الوفيات المبكرة بسبب الأمراض المزمنة.

الدروس المستفادة تم دعم المنهج الذي اتبعته سلوفينيا بواسطة هياكل صحية قوية سواء عامة أو حوكمية، إلى جانب آليات المساءلة التي تراقب النتائج وتتخذ إجراءً تصحيحياً عند الضرورة. تم إطلاق برامج الجديدة، مما أدى إلى إنشاء قاعدة أدلة قوية سهلت الحصول على تمويل مستدام، كما تم دعم التنفيذ الوطني بواسطة الفروع الإقليمية للمعهد الوطني للصحة العامة.

## 摘要

**问题:**

斯洛文尼亚的初级医疗护理模式依赖于被动型、不连贯的护理并且无法充分解决该国由非传染性疾病占主导地位的疾病负担。

**方法:**

政府为初级医疗护理制定了多学科、基于社区、以预防为主的服务提供模式。自 2000 年开始实施了一项强制性的家庭医学住院医师培训计划，并且从 2004 年起在家庭医学实践中对慢性病进行筛查和控制。建立了健康促进中心，提供团体干预以支持健康的生活方式。自 2011 年之后，引进了注册护士对慢性病进行筛查，提供咨询并管理患有稳定非传染性疾病的患者。

**当地状况:**

政府于 1992 年将斯洛文尼亚的医疗融资方案转变为基于强制性工资税的社会保险体系。尽管主要由公立社区医疗中心提供初级医疗，但该体系推动了私营医疗服务。发挥了强大的首诊作用。

**相关变化:**

虽然斯洛文尼亚的医疗支出低于欧盟 (EU) 平均水平，但截至 2013 年，斯洛文尼亚的预期寿命却高于欧盟国家的平均寿命。增长的部分原因是婴儿和五岁以下儿童的死亡率迅速下降以及因慢性病导致的过早死亡率下降更快。

**经验教训:**

斯洛文尼亚的做法得以推动的原因在于强大的公共医疗和治理结构，以及负责监控效果并在必要时采取纠正措施的问责机制。试行了新的计划，以强有力的证据为基础，有助于获得可持续融资，而全国范围内的推广则得到了国家公共医疗研究院地区分支机构的支持。

## Резюме

**Проблема:**

Модель первичного медико-санитарного обслуживания в Словении опиралась на эпизодическое обслуживание в качестве ответных мер и не справлялась с бременем болезней в стране, где преобладают неинфекционные заболевания.

**Подход:**

Правительство разработало междисциплинарную, ориентированную на профилактику модель предоставления первичного медико-санитарного обслуживания на уровне общины. В 2000 году была введена обязательная программа семейной медицины по месту жительства, а с 2004 года в практику семейной медицины были введены скрининговое обследование и мониторинг хронических заболеваний. Были созданы центры охраны здоровья, обеспечивающие групповые мероприятия для поддержки здорового образа жизни. Начиная с 2011 года проведение скрининговых обследований на наличие хронических заболеваний и консультаций, а также ведение пациентов со стабильным состоянием неинфекционных заболеваний осуществляются специальным дипломированным медицинским персоналом.

**Местные условия:**

В 1992 году правительство Словении преобразовало систему финансирования здравоохранения в систему социального страхования, основанную на обязательных налогах на заработную плату. Система позволила обеспечить частное предоставление медицинских услуг, хотя первичное медико-санитарное обслуживание в основном оказывалось общественными медицинскими центрами, финансируемыми государством. Была введена роль строгого куратора.

**Осуществленные перемены:**

Несмотря на меньшие расходы на здравоохранение, чем в среднем по Европейскому союзу (ЕС), к 2013 году ожидаемая продолжительность жизни в Словении была выше, чем в среднем по странам ЕС. Такое увеличение было отчасти обусловлено стремительным снижением младенческой и детской смертности в возрасте до пяти лет и более быстрыми темпами снижения преждевременной смертности от хронических заболеваний.

**Выводы:**

Подход Словении оказался выполним благодаря наличию надежных структур общественного здравоохранения и государственного управления, а также механизмам подотчетности, которые позволяли отслеживать результаты и принимать корректирующие меры при необходимости. Внедрялись новые программы, создающие прочную базу фактических данных, которая способствовала получению устойчивого финансирования, в то время как введение системы в действие в рамках всего государства поддерживалось региональными отделениями Национального института общественного здравоохранения.

## Introduction

The World Health Organization (WHO) defines universal health coverage (UHC) as ensuring that “all people have access to needed health services…of sufficient quality to be effective while also ensuring that the use of these services does not expose the user [to] financial hardship.”^1^ Achieving UHC is challenging for countries with a high burden of chronic diseases, for two reasons. First, most countries have a service delivery model in which episodic care is provided in response to patients presenting with symptoms of an illness: a model that is ineffective in addressing a rising burden of chronic disease. Second, patients with chronic diseases are at increased risk for high out-of-pocket payments and catastrophic health expenditure due to inadequate coverage for the necessary medications.^2^

In this paper we describe the changes that the Slovene government made, beginning in the early 1990s, to ensure financial protection for patients and improve the effectiveness of primary health care to address the country’s burden of chronic diseases more effectively.

## Local setting

Shortly after Slovenia achieved independence in 1992, law-makers passed the Health Care and Insurance Act, which transformed the country’s health financing scheme to a Bismarck-type social insurance system, based on mandatory payroll taxes for employees and employers and on pension contributions.^3^ In 2016, the scheme funded 69% of the current health expenditure, with out-of-pocket payments limited to 12% of the current health expenditure and the remainder made up of voluntary health insurance and direct government allocations for health.^4^

While the Act reformed the health financing system and enabled private provision of health services, the existing network of publicly funded community health centres remained intact with ownership transferred to municipalities. The centres provide primary care services under one roof: family medicine, paediatrics, gynaecology and dentistry practices; laboratory and diagnostic services; physiotherapy, occupational therapy, speech therapy and mental health services; community nursing; health promotion services; and selected secondary level specialist ambulatory practices. The Act also guaranteed beneficiaries free access to primary health care in facilities contracted by the national health insurance fund and the choice of primary health-care physician, who serves as the gatekeeper to specialized care.^3^

## Approach

After the passage of the Act, several initiatives were introduced to improve primary health care and address Slovenia’s burden of chronic diseases (Box 1). The Department of Family Medicine, University of Ljubljana, instituted changes to the undergraduate and graduate curriculum. A series of national health plans and strategies for specific areas of health were developed by the health ministry in collaboration with relevant stakeholders. These initiatives support continuing efforts to improve the prevention, early diagnosis and management of chronic diseases in primary care.

Box 1 **Timeline of initiatives towards comprehensive community-based primary health care in Slovenia**

**1988–1991**

Slovenia (as a part of former Yugoslavia) was one of 25 European countries participating in the Countrywide Integrated Noncommunicable Disease Intervention Programme, initiated by the World Health Organization (WHO) in 1984. The Community Health Centre in Ljubljana, representing Slovenia, formally started to participate in the programme in 1988 and established an education centre in 1991 to initiate healthy lifestyle support interventions at the primary-care level. The centre’s staff offered free courses to general practitioners and nurses interested in working according to the principles of the WHO programme. The Countrywide Integrated Noncommunicable Disease Intervention Programme became popular and many health-care teams from across Slovenia requested training as well. Lengthy negotiations with the local health administration enabled the programme movement to be extended nationally and drove screening to become a mandatory activity in family medicine.

**1994–1995**

The Department for Family Medicine, Medical Faculty, University of Ljubljana introduced family medicine as an obligatory subject in the undergraduate medicine curriculum in Slovenia. The aim was to address the need for better knowledge and new skills to enable primary health-care physicians to diagnose and treat the rising prevalence of chronic conditions seen in general practice. Courses focus on the latest evidence about the effectiveness of primary and secondary prevention of the most common chronic diseases. This initiative marks the start of a paradigm shift from the biomedical, disease-oriented model of primary health care in Slovenia to a holistic-health-oriented bio-psychosocial model of care for the entire population. The key drivers for change were general medical practitioners and national professional associations (the Slovene Family Medicine Society, part of the Slovene Medical Society).

**1995**

The Medical Faculty, University of Ljubljana, established the Department for Family Medicine to recognize the value of family medicine as a specialty with its own area of research, publications and professorship and to emphasize the need for specialized continuing medical education. The Slovene Family Medicine Society played an important role in preparing for the establishment of the university department in Ljubljana and in the recognition of family medicine practice in the professional and lay community. Other drivers for change were international contacts and researchers’ participation in international research projects, which fostered professional co-operation and gave political support for professional demands for academic recognition of family medicine.

**2000**

The Department for Family Medicine, University of Ljubljana, supported the introduction of a 4-year residency programme in family medicine that is compulsory for entry to family practice. The aim was to ensure that family physicians received adequate postgraduate training and to improve their professional position relative to other specialists, in view of the responsibility they hold as the gatekeepers to the rest of the health system. The laws to formally establish the residency programme were driven by the department, and supported by the Slovene Family Medicine Society and were the culmination of 6 years of political negotiations since the launch of university education for family physicians.

**2000–2004**

Goals and activities in the field of food safety, food supply and healthy nutrition were defined in the national health care programme 2000–2004. The Board for Food and Nutrition was established in 2000 as a consulting body to the health ministry and with responsibility for preparing a future national nutrition and food programme.

**2004**

Slovenia began the establishment of health promotion centres in community health centres and the introduction of risk-stratified cardiovascular disease and diabetes screening programmes into family medicine practices (public and private) across the country. The participation of the Ljubljana Community Health Centre in the WHO’s Countrywide Integrated Noncommunicable Disease Intervention Programme was a key driver for the change. After more negotiations, the national health insurance fund (the Health Insurance Institute of Slovenia) agreed in 2002 to ensure stable financing of the programme as the basis for the nationwide screening initiatives.

**2005**

Slovenia’s Parliament approved the national programme of food and nutrition policy 2005–2010. Recommendations from the Board for Food and Nutrition led the health ministry to formulate the Resolution on the national nutritional policy programme 2005–2010, which was adopted in the National Assembly in March 2005. The EU’s requirements on harmonization of laws and regulations for Slovenia’s accession to the EU in 2004 also contributed to the process of developing the policy.

**2010**

The Parliament approved the national programme to control diabetes mellitus 2010–2020. In response to international recommendations and national advocacy efforts, an assigned working group in the health ministry had been developing the programme for several years before finalizing a patient-centred, transparent working document for diabetes in 2009. Later that same year, the Health Insurance Institute of Slovenia joined the working group in support of the proposal. The health ministry formally approved the strategy in 2010. The strategy complements and builds on several independent health-care reforms that took place in Slovenia during the late 2000s, as described above, and is designed to strengthen existing diabetes prevention and care services.

**2010**

The Parliament approved the national cancer control programme 2010–2015, recognizing the need for a comprehensive and systematic approach to address the growing burden of cancer in the country. The programme was developed by the health ministry in accordance with international recommendations and with the participation of key national stakeholders who set goals to be achieved by 2015.

**2011**

Based on the need to improve the education of nurses, the Nurses and Midwives Association of Slovenia developed the national strategy for nursing care development and health care provision 2011–2020, which was approved by the Parliament in 2011. The strategy promotes autonomy in the nursing profession through continuous education and the development of clinical, applied and basic research, as well as an evidence-based approach to nursing. The strategy encompasses the development of nursing at all levels of the health-care system, as well as in health information, policy and legislation.

**2011**

The health ministry launched a development project in 2011 to introduce a half-time registered nurse into family medicine teams across the whole country. The initiative followed the successful introduction of a registered nurse to the family medicine practice team at the Community Health Centre of Ljubljana. Registered nurses perform screening for chronic respiratory diseases and mental health conditions, as well as counselling to individual patients and follow-up of chronic patients whose conditions are well-controlled. These registered nurses receive extensive training developed jointly by Slovenia’s National Institute of Public Health and nurses and physicians from primary and secondary care. The modular training programme is delivered by medical doctors and nurses who work in the family medicine practice.

**2014–2016**

The National Institute of Public Health, with the support of the health ministry and funding from grants from the Norwegian government led a project, starting in three pilot sites, to upgrade health promotion centres to deliver more comprehensive preventive and health promotion services on chronic diseases. Services are delivered by a broader team of multidisciplinary health professionals including dieticians, physiotherapists, kinesiologists and psychologists. An education programme to develop the skills of these professionals and enable them to deliver the new health promotion and disease prevention programmes was designed and delivered by the Institute. The scope of community nursing services was also expanded to include chronic diseases prevention services. The Institute initiated concerted efforts to coordinate with other partners delivering community services to accelerate progress on reducing health inequalities.

**2015**

The Parliament approved the national health care plan 2016–2025.^5^ The plan includes three strategies: (i) strengthening prevention and early detection of risk factors and the reduction of health inequalities at the primary care level; (ii) implementing health promotion in educational institutions, the workplace and in local environments; and (iii) integrating the management of patients with chronic diseases and conditions. Drivers for the national plan were the strong orientation of health professionals, as well as the Slovene government, towards preventive health care.

**2015**

The Parliament approved the national strategy on food, nutrition and physical activity 2015–2025, recognizing the need to integrate these actions under one holistic strategy. The continuation of the national programme of food and nutrition policy 2005–2010 was thus merged with that of physical activity. The process of programme development was led by the health ministry and involved participation by key governmental sectors, agencies, professional bodies and nongovernmental organizations.

**2016**

The national cancer control programme 2017–2021 was developed by the health ministry in cooperation with several stakeholders as a continuation of the national cancer control programme 2010–2015. The programme is a set of activities for the systematic and long-term reduction of the cancer burden in Slovenia and includes national screening programmes for breast, colorectal and cervical cancers.

**2017–2020**

The health ministry, in cooperation with the National Institute of Public Health and benefiting from funding from the EU’s structural funds, rolled-out the upgraded prevention and control programme for chronic diseases to an additional 25 community health centres. The project was developed with the aim of applying novel approaches in prevention programmes to promote health and reduce health inequalities in local communities. The programme contributes to better information about prevention, with greater involvement of the population in prevention programmes, and more accessible and appropriate preventive treatments.

**2018**

After public discussion and coordination with relevant ministries, the Parliament approved the national mental health programme 2018–2028, which was developed by the health ministry in response to the increasing burden of mental disorders. The programme strengthens mental health services and introduces multidisciplinary mental health care into primary health care. The programme enables an intersectoral, community-based and integrated approach to mental health in Slovenia.

**2019–2021**

The National Institute of Public Health, with support from the health ministry, initiated a pilot project on strengthening mental health services in primary health care. The pilot project was one of the key proposals in the national mental health programme 2018–2028 and is funded by the Health Insurance Institute of Slovenia. The project focuses on health promotion and on prevention and de-stigmatization in mental health. A network of services for mental health was established, including prevention of suicide and alcohol abuse, as well as awareness-raising, research and evaluation in mental health.

EU: European Union.

A key initiative was the establishment in 2004 of a programme for screening adults for cardiovascular diseases and diabetes. Healthy people are re-screened at 5-year intervals, while people with metabolic risk factors or newly diagnosed chronic diseases are referred to their family physician and followed up at regular intervals as specified by evidence-based protocols appropriate for their condition. In addition, health promotion centres were introduced into community health centres to provide counselling and group interventions to support healthier lifestyles for patients treated by both public and private primary health-care providers.

Beginning in 2011, a registered nurse was gradually introduced into an increasing number of family medicine practice teams and screening was extended to include chronic obstructive pulmonary disease and depression. The registered nurse also supervises the care of patients whose chronic diseases are well controlled. In 2014, health promotion centres were upgraded to deliver more comprehensive chronic disease preventive and health promotion services, delivered by a broader team of multidisciplinary health professionals including dieticians, kinesiologists and psychologists.

At the same time, the scope of community nursing services was expanded to include chronic disease prevention and registered nurses with special training (diploma) in community nursing. These community nurses receive extensive training on chronic diseases and the value of early diagnosis, treatment and healthy lifestyles. The nurses are also trained in behavioural approaches, patient-centred communication, motivational interview techniques, approaches to identifying vulnerable populations and other skills needed for good performance.

## Relevant changes

Concerted efforts towards UHC in Slovenia have led to documented improvements in a variety of performance measures. Slovenia spends less on health than the average for the 28 European Union (EU) countries. For example, in 2016 the country spent 2772 international dollars per capita on health^6^ (8.5% of its gross domestic product, GDP).^7^ This compared with an average health spending of 3846 in international dollars per capita (9.9% of GDP) in the EU.^6^ Yet at the same time Slovenia has achieved one of the lowest rates of catastrophic health expenditure in the WHO European Region^2^ (historical data on catastrophic spending are not available).

**Fig. 1:**
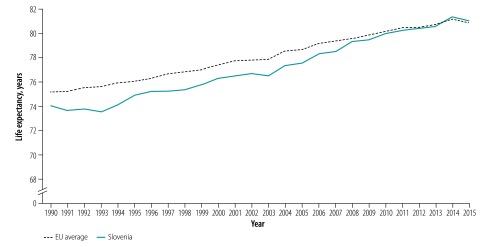
Trends in life expectancy, Slovenia and EU, 1990–2015

Life expectancy in Slovenia has increased at a faster rate than the EU average and in 2013 began to exceed the EU average (Fig. 1).^8^ The increase is in part due to Slovenia’s low and more rapidly declining mortality in young children. Infant mortality rate fell from 8.8 deaths per 1000 live births in 1990 to 1.7 per 1000 live births in 2018 (corresponding to an annualized decline of 5.7%), compared with a decline in the EU average from 9.9 deaths per 1000 live births to 3.3 deaths per 1000 live births in the same period (annualized decline of 3.8%).^9^ Among children younger than 5 years of age, mortality in Slovenia decreased from 10.4 deaths per 1000 live births in 1990 to 2.1 deaths per 1000 live births in 2018 (annualized decline of 5.6%), compared with a decline in the EU average from 11.9 deaths per 1000 live births to 4.0 deaths per 1000 live births in the same period (annualized decline of 3.8%).^10^ Another factor in greater life expectancy is the 26% greater annualized rate of decline in the probability of dying from cardiovascular disease, cancer, diabetes and chronic obstructive pulmonary disease. For example, between 2000 and 2018 Slovenia experienced an annualized decline of 1.4% per year, compared with the EU’s 1.1% (our calculations based on World Development indicators).^11^

EU: European Union

Note: The average for the 28 countries of the European Union.

Source: European Health Information Gateway.^6^

Estimates of premature mortality (years of life lost) due to metabolic risk factors have also decreased at a faster rate in Slovenia than in the EU between 2003 and 2017 (by 1.7% versus 1.3% per year in Slovenia and the EU, respectively; our calculations based on the Global Burden of Disease Study, 2017).^12^ These estimates provide further evidence that Slovenia’s focus on health promotion and disease prevention and management in primary health care is having a positive impact.

The Health Care Access and Quality Index measures access to and quality of a country’s health services based on mortality data of 32 diseases that should not lead to death in the presence of effective and safe health care.^13^ Slovenia scored 91 out of 100 in 2016, up from 74 in 1990, ranking it 21 out of 195 countries.^13^ While Slovenia’s score has not yet caught up with the average of western European countries (score 79 in 1990 and 93 in 2016), it exceeded the average of central European countries (score 59 in 1990 and 81 in 2016; data are not available for the EU average).^13^ Collectively, these findings suggest the effectiveness of Slovenia’s efforts to develop its primary health-care system.

## Lessons learnt

Due to continuing reforms and strengthening of primary health care, Slovenia comes close to providing the type of person-centred, integrated care, which the Astana Declaration of 2018 argues is “the most inclusive, effective and efficient approach to enhancing people’s physical and mental health.”^14^ Many factors have contributed to the government’s achievements. At the national level, development strategies and national health plans prioritized primary health care and ensured adequate funding to achieve the country’s goals. Professional organizations, particularly the Slovene Family Medicine Society and involvement in international collaborations were key drivers for change in the continuing efforts to strengthen primary care.

Slovenia’s public health system, supported by the health’s ministry’s Directorate of Public Health, has been an essential part of the design of new disease prevention and health promotion programmes and their successful integration into primary health care (Box 2). New programmes were first piloted in primary health-care facilities in a few municipalities, often as part of an EU-funded project. These pilot facilities provided the resources and staff needed to identify and address the inevitable challenges before the programmes were rolled out across the country. Strong evaluation mechanisms generated evidence of the programmes’ impact, facilitating sustainable financing from the national health insurance fund. In addition, the regional branches of Slovenia’s National Institute of Public Health supported community health centres when the programmes were rolled out, another element that helped to ensure sustainability.

Box 2 **Summary of main lessons learnt**

- Multidisciplinary teams in health promotion centres, integrated into Slovenia’s community health centres, ensure the delivery of comprehensive and integrated health services that include programmes to prevent and manage chronic diseases.
- Supportive national health plans and strong institutional capacity at Slovenia’s health ministry and national public health institute were important for the design and implementation of new public health programmes in community health centres.
- Effectively implemented pilot schemes helped to create a strong evidence base for the effectiveness of these programmes and facilitated sustainable financing for national roll-out.

These achievements are at risk, however. Many publicly employed family physicians are unhappy, periodically threatening to strike or resign. The discontent is due to several factors: the rising administrative burden imposed by the national health insurance fund; the difficult-to-use electronic patient record system; the outdated governance model for public primary-care facilities that limits the authority of their managers; and increases in the workloads of salaried primary care physicians without additional pay. Strategies to address these challenges have been included in successive national health plans, but have not been implemented. That these plans have not succeeded suggests a need to strengthen the institutional capability of Slovenia’s health ministry to design and implement the necessary structural reforms to address the root causes of challenges faced by the primary health-care system.

Slovenia’s experience may serve as inspiration for countries wishing to improve their primary health-care services. A person-centred, integrated primary care model that emphasizes health promotion and disease prevention programmes is valuable for countries who need to tackle their burden of chronic diseases more effectively and progress towards UHC. Outcome-focused, user-friendly electronic patient record systems are essential for continuous, data-driven quality improvement at the facility level. Countries need to develop a strong state capability to monitor the health system’s performance and take corrective action when needed. 
